# Jejuno-ileal diverticulitis: A disorder not to underestimate

**DOI:** 10.1016/j.ijscr.2019.04.015

**Published:** 2019-04-16

**Authors:** Alice Maria Ramistella, Massimo Brenna, Fabrizio Fasolini, Marco De Monti

**Affiliations:** EOC – Beata Vergine Regional Hospital, Department of Surgery, CH 6850, Mendrisio, Switzerland

**Keywords:** Case report, Emergency surgery, Diverticulitis, Jejuno-ileal diverticula, Adhesiolisis, Peritonitis

## Abstract

**Introduction:**

Jejuno-ileal diverticulitis is an uncommon, acquired clinical entity, with higher prevalence among patients aged between 60 and 70. The condition is usually silent and has been regarded as relatively innocuous. Sometimes patients complain chronic vague symptoms like malabsorption, pain or nausea, that easily lead to misdiagnosis. Acute complications are rare, however, they have been reported and can result in major surgery and high overall mortality.

**Case report:**

We are presenting a case of a 67-year-old patient who presented to our department with abdominal pain and signs of peritonitis. The CT scan displayed an inflammatory mass with a fair amount of free liquid in the abdomen, as well as multiple diverticula at different levels of the intestine. The patient had to underwent immediate surgery, during which a resection of 25 cm jejunum and 80 cm of ileum has been performed.

**Conclusion:**

Jejuno-ileal diverticula are a very uncommon finding that can present formidable challenges in diagnosis and treatment. The course can be completely asymptomatic, however, in rare cases, the condition can lead to severe complications that often require surgery. Multi detector CT (MDCT) with intravenous contrast should always be the modality of choice for investigating a suspect of small bowel diverticula. There are no specific guidelines respect the management of jejuno-ileal diverticulitis, nevertheless, in the acute setting, bowel resection is the treatment of choice.

As well as setting out the rarity of this case, our work intends to review the current literature regarding the epidemiology, natural history, diagnosis and management of jejuno-ileal diverticula

## Highlights

• In the paper a rare case of complete and well documented jejunal diverticulitis complicated with perforation and peritonitis is described.
• Interesting radiological and intraoperative imaging are attached.
• A carefull and recent literature review has been performed in order to discuss diagnosis and management of jejuno-ileal diverticula and our clinical behavior.
• From the discussion emerges that the possibility of the presence of small bowel diverticula must be considered in case of occult bleeding non-indentifiable with gastroscopy or colonoscopy.
• Therapeutic behavior is suggested in case of acute peritonitis due to jejunal diverticula or in case of incidental diagnosis.

## 1 Introduction

This work has been reported in line with the SCARE criteria [1].

Jejuno-ileal diverticula are an extremely rare condition, usually found incidentally in males in their sixth or seventh decade (male/female ratio of 2:1). The incidence ranges from 0.03% to 8.0% on autopsy series, and between 0.02% and 7% on patients studied with contrast examinations [2]. The majority of jejuno-ileal diverticula occurs along the mesenteric border of the small bowel, being usually multiple and organized in clusters.

The probability of finding diverticula decreases towards the ileocecal valve [3]. 10% of patients diagnosed with jejuno-ileal diverticula will develop complications [2]. Complications as perforations, adhesions, fistula, peritonitis have the greater frequency, while massive lower gastrointestinal bleeding is reported to be more uncommon, with only 50 cases reported in literature [2,4]. We report herein a case of a 67-year-old man who was known for gastrointestinal bleeding of uncertain origin, later diagnosed with diverticulitis of the small bowel and presented to our department after two months because of an acute abdomen due to perforated jejuno-ileal diverticula.

## 2 Case report

A 67-year-old Caucasian man presented to the emergency room because of a 4 days’ history of abdominal pain, with one episode of vomiting.

The patient’s past medical history was significant for colonic diverticulosis and an episode of gastrointestinal bleeding one year before. The event had been investigated by two different gastroscopies, a colonoscopy and a MDCT, which produced inconclusive results. Ten months later he was newly admitted because of abdominal pain and fever at 38.0 C°, with valid urination and defecation. A CT of the abdomen was performed, which confirmed the colonic diverticulosis and revealed the presence of multiple diverticula of the small intestine, fat stranding, signs of inflammation as well as a small amount of free liquid in the abdomen. The patient was hence diagnosed with jejunal diverticulitis and managed conservatively with intra-venous antibiotics, with an apparent complete recovery.

**Fig. 1:**
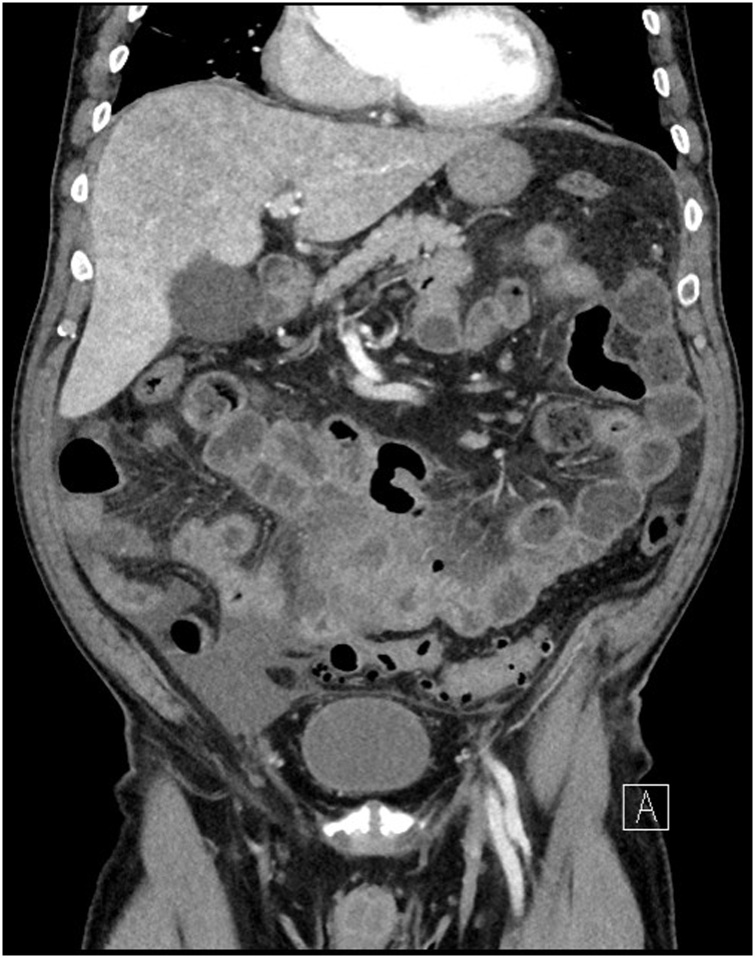
Coronal image from a contrast enhanced CT scan showing extensive bowel wall thickening and mesenteric fat stranding with free liquid in the right pelvic area.

**Fig. 2:**
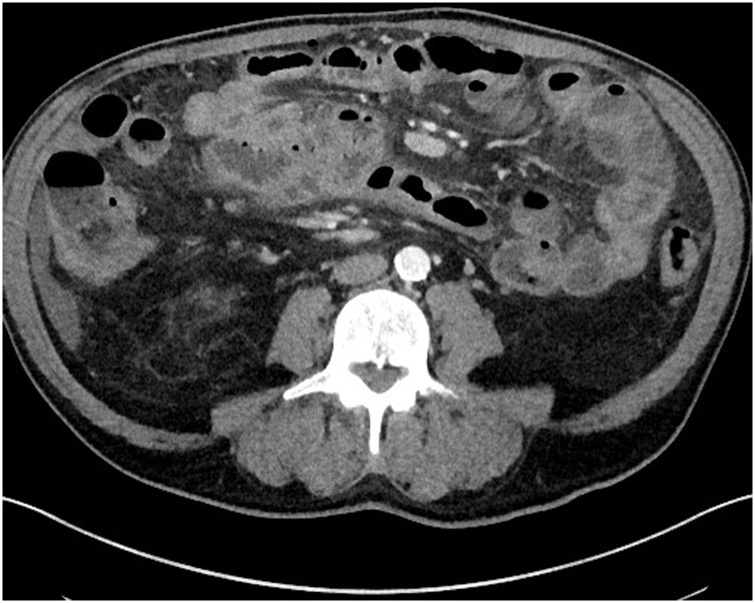
Axial CT scan showing adhesions between thick-walled segments of the small bowel and fat stranding.

He re-presented to the emergency department two months later with acute abdominal pain. The pain was described as severe and constant, localized mainly in the lower abdomen with clinical signs of peritonitis. No change in bowel habits nor urinary symptoms were complained. His vital signs were stable, with a temperature of 37.2 C°; he appeared fully oriented and not in any acute distress. Laboratory examination reported a hemoglobin of 121 g/dL, a WBC count of 12.2 × 10E9/L and a CRP of 249 mg/L. Other laboratory data were within normal limit. An abdominal and pelvic contrast-enhanced computed tomography, with administration of oral contrast, was performed. Jejunum and ileum showed several diverticula as well as an inflammatory thick-walled mass involving different loops of the intestine. In addition, free fluid in the abdomen and a small amount of subdiaphragmatic air were reported (Fig. 1, Fig. 2). On the basis of these findings, the diagnosis of perforated diverticulitis was hereby proposed.

**Fig. 3:**
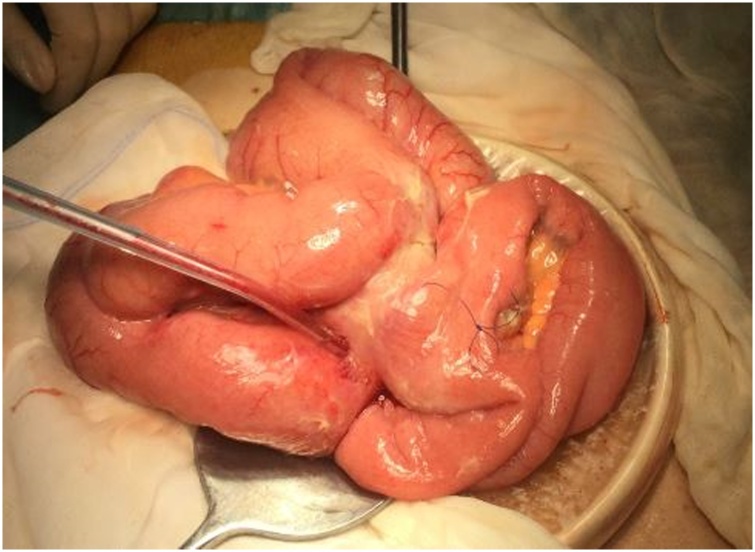
Intraoperative image: strong adhesions between the middle distal tract of the jejunum and the middle distal tract of ileum.

**Fig. 4:**
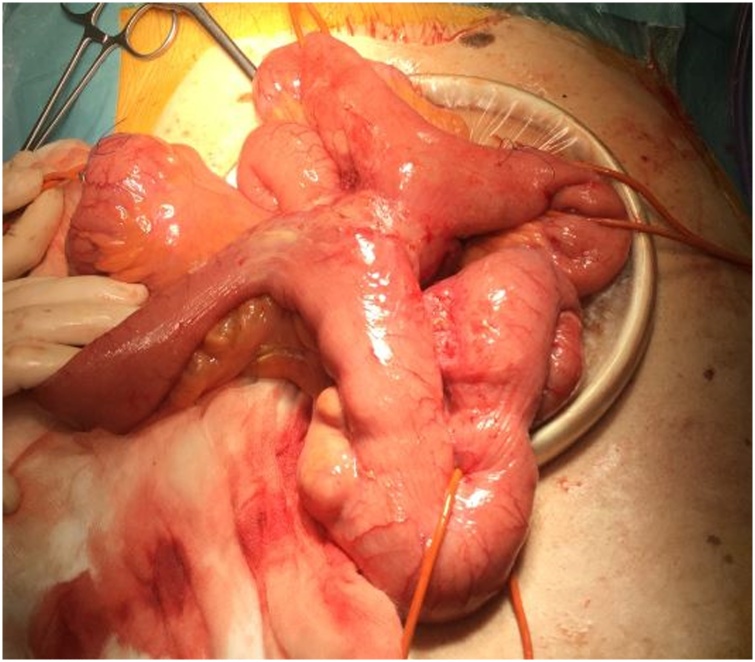
Intraoperative image: the segments of jejunum and ileum involved by adhesions were connected by an intestinal loop free of signs of diverticulosis.

The patient underwent a diagnostic laparoscopy which revealed plenty of purulent yellowish liquid collected in the right abdomen and a conglomerate of intestinal inflamed loops. We hence decided to convert immediately the procedure to laparotomy. Large multiple diverticula were found covering a section of small intestine approximately 2.5 m long, without signs of obvious macro perforation. Among the middle distal tract of the jejunum and the middle distal tract of ileum, strong adhesions were identified (Fig. 3). The involved segments of jejunum and ileum were connected by an intestinal loop free of signs of diverticulosis (Fig. 4). There were no signs of bowel ischemia. Adhesiolisis was partially carried out, however, because of difficulties associated with the procedure, we opt to perform a double enterectomy, removing only those segments involved in the intestinal conglomerate and deeply affected by the pathology. Roughly 25 cm of ileum and 80 cm of jejunum were resected. Bowel continuity was restored with an ileo-ileal and a jejuno-jejunal anastomosis. Almost 700 cl of pus were drained and the peritoneal cavity was washed with 10 L of saline solution. The postoperative recovery was uneventful and the patient was discharged 8 days later. There were no signs of malignancy in the resected intestine.

## 3 Discussion

Sommering was the first to report acquired jejuno-ileal diverticula in 1794 [5]. These lesions are considered as herniation of the mucosa, submucosa and serosa through the muscular layer of the bowel. Small intestine diverticula are thin-walled and usually found organized in clusters on the mesenteric border of the bowel [6]. The etiology was suggested in 1968 by Cock and Zeno, who stated that diverticula occur where the vasa recta blood vessels penetrate the mesentery area, generating a structural weakness [6,7]. Others believe that they are the result of motility disorder or ineffectiveness in contraction of the muscular intestinal wall [6]. Small bowel diverticula have a bigger incidence in elderly males and they commonly affect the proximal jejunum (75%). Distal jejunum and ileum are co-affected in 20% and 5% of the cases, while 30%–75% of the patients present coexistent diverticula in the colon [8]. Approximately 0.07–1.0% of the population is reported to have jejuno-ileal diverticula; however, it is believed that the numbers are probably an underestimation of the true incidence [9]. The condition is usually clinically silent, as a matter of fact, it has been estimated that only 29% of the patients complains symptoms or signs, usually related to malabsorption and therefore associated to dyspepsia, abdominal discomfort and anemia [5]. Between patients with duodenal and jejuno-ileal diverticula, 10% of them will go through complications, especially those with jejuno-ileal diverticula, that, in comparison with individuals with duodenal diverticula, are four times more likely to have a general complication and about 18 times more likely to develop a perforation [9]. Acute complications are related to the inflammation of the mucosa, that leads to its perforation and subsequent abscess, massive hemorrhages or intestinal obstruction. Gastrointestinal hemorrhage can often be the presenting symptom of jejuno-ileal diverticula, however, even if MDCT as well as scan with radio-targeted erythrocyte might be of help, properly identify the correct diagnosis remains challenging. If diverticula have been recognized, a careful investigation of the small bowel is mandatory, and usually, in presence of lesions, the resection of the involved tract of the intestine with primary anastomosis is the treatment of choice [11].

Perforation of jejunal diverticula is a severe complication that occurs in 2.3%–6.4% of the cases, and it is often due to inflammation and subsequent necrosis of the mucosa. Even micro-perforations can produce a local inflammatory process. The bowel response results in the formation of an abscess, with migration of the surrounding intestinal tracts and soft tissues, causing adhesions between loops. Perforated jejunal diverticula can further complicate with fistulas between intestinal loops, abdominal wall abscesses and suppurative pyelophlebitis [10,11].

Diagnosis of jejuno-ileal diverticula is arduous, even in presence of symptomatic complications, therefore a highly clinical suspicion on the part of physicians is required. Endoscopic methods and abdominal ultrasounds identify with difficulties pathology of the small bowel. Abdominal or chest radiograph can show signs of perforation, evidences of intestinal obstruction or ileus. MDCT may really help to recognize the condition, exposing signs of inflammation such as fat stranding, free liquid and air in the abdomen, in presence of out-pouching lesions with thickened walls [12]. However these procedures often produce inconclusive results. An explorative laparoscopy, in presence of high clinical suspect and acute presentation, should be always performed, since it enables an accurate diagnosis to be made. It is important to avoid delays in diagnosis; given that it can be fatal. In fact, it is estimated that mortality rate for patients with jejunal diverticulitis ranges from 0 to 5%, but in case of perforation the incidence is reported to be as high as 40% [13].

No consensus has been reached about therapeutic management of patient with symptomatic jejunal diverticular disease. When only mild inflammation signs are shown, bowel rest and use of antibiotics can be attempted [10]. In case of complication such as massive gastro-intestinal bleeding, perforations or abscesses, guidelines are controversial and the management has to be tailored on patient.

When the perforation causes localized peritonitis but the patient has stable vital signs, conservative treatment with a percutaneous Ct-guided aspiration of the intraperitoneal collection can be performed with good results, avoiding the need of surgery [12,14]. Lesser procedures as diverticulectomy, simple closure or invagination of the diverticulum are associated with a three time higher mortality rate [10,12,14] and are only recommended when the perforated diverticulum is situated next to the duodeno-jejunal flexure, because of difficulties in managing anastomotic complications at this region [14]. However, the treatment of choice for complicated jejuno-ileal diverticulitis causing generalized peritonitis is prompt laparotomy with segmental small bowel resection followed by primary anastomosis. This is especially recommended when bleeding, obstruction or signs of perforations are shown [10,[12], [13], [14]]. As a matter of fact, Wilcox and Clayton in 1988 stated that retrospective studies showed that up to 15% of patients with jejunal diverticulosis will require intestine resection for complications such as perforation and diverticulitis [15]. On the other hand, it is demonstrated that, although in many occasions surgery seems to represent the best option for the patient, when possible, resection has to be limited, due to the risk of bowel syndrome as well as recurrences of the diverticula [13,14]. Diverticula discovered incidentally during laparoscopic or laparotomic procedures do not require any interventions [14]. Overall mortality rate after general surgery is 24% and 14% after the enterectomy of the involved intestinal loops, being poor prognostic factors the advanced age of the patients as well as delayed diagnosis and intervention [10,14].

## 4 Conclusions

Even if jejuno-ileal diverticula are extremely rare and usually do not request surgical treatment, they should not be regarded as an insignificant finding, given that when these lesions produce complications, the consequences can be serious. In conclusion, because of the morbidity and mortality associated with a delay diagnosis, it is important to raise awareness of the fact that a presentation of abdominal pain could be due to jejuno-ileal diverticulitis, especially in older population with a history of gastrointestinal hemorrhage of unknown origin.

## Conflicts of interest

All Authors disclose any financial and personal interest

## Sources of funding

This study has not received any sponsorship

## Ethical approval

This is a case report and not a retrospective or perspective study and doesn’t need ethical approval

## Consent

Written informed consent has been obtained

## Author contribution

A.M. Ramistella: Writer of paper

M. Brenna: Study concept ideator

F. Fasolini: Supervisor

M. De Monti: Data check and literature research – Corresponding author

## Registration of research studies

We present a case report

## Guarantor

Marco De Monti M.D.

## References

[1] Agha R.A., Borrelli M.R., Farwana R., Koshy K., Fowler A., Orgill D.P., For the SCARE Group (2018). The SCARE 2018 statement: updating consensus surgical CAse REport (SCARE) guidelines. Int. J. Surg..

[2] Transue D.L., Hanna T.N., Shekhani H., Rohatgi S., Khosa F., Johnson J.O. (2017). Small bowel diverticulitis: an imaging review of an uncommon entity. Emerg. Radiol..

[3] Miller R.E., McCabe R.E., Salomon P.F., Knox W.G. (1970). Surgical complications of small bowel diverticula exclusive of Meckel’s. Ann. Surg..

[4] Staszewicz W., Christodoulou M., Proietti S., Demartines N. (2008). Acute ulcerative jejunal diverticulitis: case report of an uncommon entity. World J. Gastroenterol..

[5] Singal R., Gupta S., Airon A. (2012). Giant and multiple jejunal diverticula presenting as peritonitis a significant challenging disorder. J. Med. Life.

[6] Ejaz S., Vikram R., Stroehlein J.R. (2017). Non-meckel small intestine diverticulitis. Case Rep. Gastroenterol..

[7] Edwards H.C. (1954). Intestinal diverticulosis and diverticulitis. Ann. R. Coll. Surg. Engl..

[8] Singh S., Sandhu H.P., Aggarwal V. (2011). Perforated jejunal diverticulum: a rare complication. Saudi J. Gastroenterol..

[9] Lacz N.L., Zurlo J.V. (2010). Small bowel diverticulitis: an often overlooked cause of acute abdomen. Emerg. Radiol..

[10] de Bree E., Grammatikakis J., Christodoulakis M., Tsiftsis D. (1998). The clinical significance of acquired jejunoileal diverticula. Am. J. Gastroenterol..

[11] Taylor M. (1969). Massive hemorrhage from jejunal diverticulosis. Am. J. Surg..

[12] Butler J.S., Collins C.G., McEntee G.P. (2010). Perforated jejunal diverticula: a case report. J. Med. Case Rep..

[13] Kassir R., Boueil-Bourlier A., Baccot S., Abboud K., Dubois J., Petcu C.A., Boutet C., Chevalier U., Montveneur M., Cano M.I., Ferreira R., Debs T., Tiffet O. (2015). Jejuno-ileal diverticulitis: etiopathogenicity, diagnosis and management. Int. J. Surg. Case Rep..

[14] Syllaios A., Koutras A., Zotos P.A., Triantafyllou E., Bourganos N., Koura S., Liakos A. (2018). Jejunal diverticulitis mimicking small bowel perforation: case report and review of the literature. Chirurgia (Bucur).

[15] Cunningham S.C., Gannon C.J., Napolitano L.M. (2005). Small-bowel diverticulosis. Am. J. Surg..

